# The *NF1* somatic mutational landscape in sporadic human cancers

**DOI:** 10.1186/s40246-017-0109-3

**Published:** 2017-06-21

**Authors:** Charlotte Philpott, Hannah Tovell, Ian M. Frayling, David N. Cooper, Meena Upadhyaya

**Affiliations:** Division of Cancer and Genetics, Institute of Medical Genetics, Cardiff University, Heath Park, Cardiff, CF14 4XN UK

**Keywords:** NF1, Sporadic tumours, Somatic mutations, Cancer, Melanoma, Lung cancers, Glioblastoma, Leukaemia, Breast cancer, Phaeochromocytoma

## Abstract

**Background:**

Neurofibromatosis type 1 (NF1: Online Mendelian Inheritance in Man (OMIM) #162200) is an autosomal dominantly inherited tumour predisposition syndrome. Heritable constitutional mutations in the *NF1* gene result in dysregulation of the RAS/MAPK pathway and are causative of NF1. The major known function of the *NF1* gene product neurofibromin is to downregulate RAS. NF1 exhibits variable clinical expression and is characterized by benign cutaneous lesions including neurofibromas and café-au-lait macules, as well as a predisposition to various types of malignancy, such as breast cancer and leukaemia. However, acquired somatic mutations in *NF1* are also found in a wide variety of malignant neoplasms that are not associated with NF1.

**Main body:**

Capitalizing upon the availability of next-generation sequencing data from cancer genomes and exomes, we review current knowledge of somatic *NF1* mutations in a wide variety of tumours occurring at a number of different sites: breast, colorectum, urothelium, lung, ovary, skin, brain and neuroendocrine tissues, as well as leukaemias, in an attempt to understand their broader role and significance, and with a view ultimately to exploiting this in a diagnostic and therapeutic context.

**Conclusion:**

As neurofibromin activity is a key to regulating the RAS/MAPK pathway, *NF1* mutations are important in the acquisition of drug resistance, to BRAF, EGFR inhibitors, tamoxifen and retinoic acid in melanoma, lung and breast cancers and neuroblastoma. Other curiosities are observed, such as a high rate of somatic *NF1* mutation in cutaneous melanoma, lung cancer, ovarian carcinoma and glioblastoma which are not usually associated with neurofibromatosis type 1. Somatic *NF1* mutations may be critical drivers in multiple cancers. The mutational landscape of somatic *NF1* mutations should provide novel insights into our understanding of the pathophysiology of cancer. The identification of high frequency of somatic *NF1* mutations in sporadic tumours indicates that neurofibromin is likely to play a critical role in development, far beyond that evident in the tumour predisposition syndrome NF1.

## Background

Neurofibromatosis type 1 (NF1: Online Mendelian Inheritance in Man (OMIM) #162200) is an autosomal dominantly inherited tumour predisposition syndrome. Affecting 1/3000–4000 individuals worldwide, it results from constitutional mutations of the *NF1* gene, lo\cated on the long arm of human chromosome 17 [1–4]. A variety of characteristic clinical features are associated with NF1, including hyperpigmentary abnormalities of the skin (café-au-lait macules (CALMs) and inguinal/axillary freckling, iris hamartomas (Lisch nodules) and the growth of benign peripheral nerve sheath tumours (neurofibromas) in the skin. Neurofibromas can be divided into several different subtypes and are associated with a variety of clinical complications. Cutaneous neurofibromas are small, discrete dermal tumours observed in most, but not all, adult NF1 patients [5]. The generally much larger plexiform neurofibromas (PNFs), a more diffuse tumour type, are present in 30–50% of NF1 patients. Importantly, some 10–15% of these benign PNFs subsequently develop into aggressive malignant peripheral nerve sheath tumours (MPNSTs) which are the main cause of morbidity in NF1 [6–8]. A number of other tumours are also associated with NF1, including optic gliomas, juvenile myelomonocytic leukaemia (JMML), benign or malignant phaeochromocytomas, gastrointestinal stromal tumours, glomus tumours, juvenile xanthogranulomas, rhabdomyosarcomas and lipomas.

*NF1* is a tumour suppressor gene; in order for a particular cell to become cancerous, both alleles of a tumour suppressor gene must be mutated. This concept, known as the ‘two-hit’ hypothesis, was first proposed by Knudson, and the majority of NF1-associated tumours exhibit biallelic inactivation of *NF1* [9, 10].

The *NF1* gene is spread over a large locus (350 kbp) at 17q11.2. It contains 61 exons, including four alternatively spliced exons, and is transcribed into a 12 kbp messenger RNA (mRNA) containing an open reading frame of 8454 nucleotides [11]. Curiously, intron 27b, the largest intron of *NF1* at 61 kbp, contains three embedded genes, *OMGP*, *EVI2B* and *EVI2A*, that are all transcribed in the opposite orientation to *NF1* but whose protein products appear to have little or no interaction with neurofibromin [11].

### Neurofibromin: the *NF1* gene product

Neurofibromin is a 2818 amino acid, multidomain protein. Although ubiquitously expressed, its highest levels are to be found in cells of the central nervous system (CNS), where it is often found in association with tubulin. Neurofibromin is a member of a large family of evolutionarily conserved proteins: the mammalian Ras-GTPase-activating protein (GAP)-related proteins, and its most highly conserved region is the centrally located GAP-related domain (GRD), which is encoded by exons 20–27a. The best understood function of neurofibromin is its role in tightly regulating cellular levels of activated RAS proteins. All RAS proteins exist in two cellular states, the majority being found in their inactive GDP-bound form, with only a very small fraction present in their metabolically active GTP-bound form. Only in their GTP-bound form are RAS proteins able to upregulate the many downstream effector proteins that form part of the RAS/RAF/MAPK signalling pathway [12–16]. The key role of neurofibromin is to downregulate the activated GTP-bound RAS by stimulating the low intrinsic GTPase activity of the RAS proteins themselves, thereby promoting the conversion of active RAS-GTP into its inactive RAS-GDP state. Hence, any loss of neurofibromin functionality, due to inactivating mutations in *NF1*, will result in sustained intracellular levels of active RAS-GTP, resulting in prolonged activation of the RAS/RAF/MAPK signalling pathway and ultimately a loss of growth control and increased cellular proliferation.

Increased active RAS-GTP levels also stimulate the PI3K/AKT/mTOR signalling pathway which protects cells from apoptosis. In the absence of functional neurofibromin, the pathway can become constitutively activated resulting in an increase in cell proliferation and survival. The RAF/MAPK and PI3K/AKT pathways both activate mTOR signalling, a process found to be highly regulated in neurofibromas whereby mTOR pathway activation occurs in the absence of growth factors, in both NF1 tumours and neurofibromin-deficient cultured cells. Indeed, the mTOR pathway is constitutively activated in neurofibromin-deficient primary cells and tumours, and is regulated by phosphorylation and inactivation of the *TSC2*-encoded protein tuberin by AKT, ERK and RSK [13, 17]. It has also been suggested that increased RAS activity in brain cells may be associated with NF1-related learning deficiencies; it may result in long-term impairment as a result of increased GABA-mediated inhibition [18]. Neurofibromin levels and therefore Ras signalling can also be affected by mechanisms other than *NF1* mutation including ubiquitination [19].

Neurofibromin is known to associate with a large number of proteins, including tubulin, kinesin, protein kinases A and C, syndecan, caveolin, cytokeratin intermediate filaments and the amyloid precursor protein, although the biological significance of these protein-protein interactions is largely unknown. The diversity of protein associations does however emphasize the point that neurofibromin is likely to have many functions other than merely functioning as a GAP protein [14, 20]. Nonetheless, to date, only the function of the GAP-related domain of neurofibromin is fully understood, so it is to be hoped that new molecular studies will reveal additional functional properties of neurofibromin [21].

### Mutation analysis of the *NF1* gene

The germline mutation rate of *NF1* is some 10-fold higher than that observed for most other inherited disease genes, with more than half of NF1 cases attributed to de novo mutations [7]. Currently, over 2600 different inherited mutations in *NF1* have been reported in the Human Gene Mutation Database (HGMD®) as a cause of NF1, varying in size from large genomic deletions spanning several megabases to single base-pair substitutions that alter an encoded amino acid or the function of a splice site [22–26]. There is, however, no evidence of any localized mutation clustering within *NF1*. Whilst the constitutional *NF1* mutational spectrum is well defined with missense/nonsense (27.7%), splicing (16.3%), microdeletions (26.9%), microinsertions (11.1%), indels (2.0%), gross deletions (>20 bp; 13.3%), gross insertions (>20 bp; 2.0%), complex rearrangements (0.6%) and a couple of putative regulatory mutations, there is no evidence of any localized mutation clustering within *NF1* [27, 28]. The majority (>80%) of constitutional *NF1* mutations are inactivating, predicted to result in almost complete absence of the transcript or protein [25]. Approximately 5–10% of all heritable *NF1* mutations involve gross DNA alterations, mainly genomic deletions spanning the whole gene and flanking region, as well as intragenic multi-exon rearrangements [29]. Constitutional mutations have not been identified in any of the four alternatively spliced exons in research studies, but this may be due to ascertainment bias, as the majority of clinical laboratories that analyse *NF1* do not screen these alternative exons for mutations.

A subset of the many *NF1* splicing mutations, i.e. deep intronic mutations, result in the creation of novel acceptor/donor splice sites. These may give rise to the inclusion of a novel cryptic exon into the transcribed mRNA, leading to the production of an aberrant neurofibromin protein. Such mutations account for ~2% of all reported constitutional *NF1* mutations [30].

To date, only three NF1 families with gonadal or germline mosaicism have been reported [31–33]. In such families, only a small proportion of the germ cells, whether sperm or ova, will carry the new *NF1* mutation, but this can nevertheless result in more than one affected child being produced by clinically normal parents [34].

A major challenge for clinicians and geneticists dealing with NF1 is the successful identification and characterization of causative *NF1* mutations in their patients. This problem relates to a number of features of the *NF1*, including its large genomic size (~350 kbp) and complexity (61 exons), the absence of any obvious mutational hotspots or recurrent mutations, and the wide spectrum of mutation types observed. Indeed, the lack of mutational clustering and the paucity of recurrent mutations necessitates analysis of the entire *NF1* gene in the search for potential pathogenic mutations. Furthermore, given the broad spectrum of known *NF1* mutations, no single mutation detection test can, as yet, successfully identify all such mutation types [35]. Furthermore, some 30% of all *NF1* mutations are predicted to cause aberrant splicing, and for this reason, the analysis of both RNA and DNA from patients in mutation screening protocols is clearly required [25]. Whilst the majority of *NF1* splicing mutations occur within consensus acceptor and donor splice site sequences, a number of missense, nonsense, and even ‘silent’ mutations may also result in aberrant splicing, which are often only identifiable by screening a patient’s RNA [25]. As well as the challenges in collection and analysis of patient mRNA, a frequent issue is the difficulty in interpreting the clinical diagnostic significance of putative *NF1* missense mutations, as this may require a family segregation study and/or in vitro functional analysis to determine the pathogenicity (or otherwise) of the variant in question [25, 36].

Furthermore, many highly homologous *NF1* pseudogene sequences are scattered throughout the human genome and can often interfere with PCR-based diagnostic tests. This emphasizes the need for the careful selection of PCR primers to avoid non-specific amplification of these pseudogene sequences.

The spatial distribution of *NF1* microdeletions is strongly influenced by the presence of a number of low-copy repeats (LCRs) spanning the 17q11.2 region that encompasses the *NF1* gene. Indeed, studies into *NF1* microdeletions have provided a general model to understand the different mutational mechanisms underlying large genomic rearrangements associated with inherited diseases [37].

The *NF1* mutation detection rate in classical NF1 patients can be up to 95%. However, somatic mutation detection is more challenging, largely because of the cellular heterogeneity which is characteristic of tumour tissue [38]. Mutations in multiple genes encoding the components of the RAS/MAPK pathway predispose patients to develop clinical features that overlap with those of NF1, e.g. Legius syndrome, Noonan syndrome inter alia, and the majority of these conditions are associated with tumours [39].

### Tumour biology

All cancers originate from a single cell that starts to behave abnormally due to acquired somatic mutations in its genome. These somatic mutations may be the consequence of impaired DNA replication machinery, exogenous or endogenous mutagen exposures, enzymatic modification of DNA or defective DNA repair.

A subset of these somatic changes, termed ‘driver mutations’, confer a selective growth advantage and are implicated in cancer development, whereas the remainder are considered to be ‘passengers’ [40]. The Cancer Genome Atlas (TCGA), International Cancer Genome Consortium (ICGC), Catalogue of Somatic Mutations in Cancer (COSMIC) and cBioPortal for Cancer Genomics collectively represent the results of large-scale sequencing of cancers, thereby capturing many of the genomic alterations driving malignancy [41–44]. The cBio Cancer Genomics Portal is an open-access resource for the interactive exploration of multidimensional cancer genomics data sets, currently providing access to data from more than 5000 tumour samples from 147 cancer studies [44–46]. It contains data on somatic *NF1* mutations in different types of tumour including melanoma (desmoplastic, skin cutaneous and uveal), breast carcinoma, neuroendocrine prostate cancer, glioblastoma, lung adenocarcinoma and squamous cell carcinoma, urothelial carcinoma, uterine carcinoma, adenoid and ovarian serous cystadenocarcinoma, paraganglioma, phaeochromocytoma, pancreatic cancer, adrenocortical carcinoma, stomach adenocarcinoma, sarcoma, oesophageal cancer, rhabdomyosarcoma and many more. In this review, we detail the frequency of somatic *NF1* mutations in many non-NF1-associated sporadic cancers including melanoma, glioblastoma, neuroblastoma, breast cancer, ovarian serous carcinoma, paraganglioma and phaeochromocytoma, lung adenocarcinoma, lung squamous cell carcinoma, bladder, colorectal and leukaemia. Further, it is anticipated that with the advent of powerful sequencing technologies, combined with precise microdissection of tissue, somatic *NF1* mutations will be identified in additional tumour types. Somatic *NF1* mutations are important not only because they may be drivers but also because they may contribute to resistance to therapy [47]. Elucidation of the mutational landscape of somatic *NF1* mutations in a large number of sporadic tumours, their role in the initiation and progression of tumours and how they can confer resistance or sensitivity to a therapeutic intervention may provide further insight into the mechanisms underlying tumour development and ultimately aid the development and targeting of therapies.

**Fig. 1:**
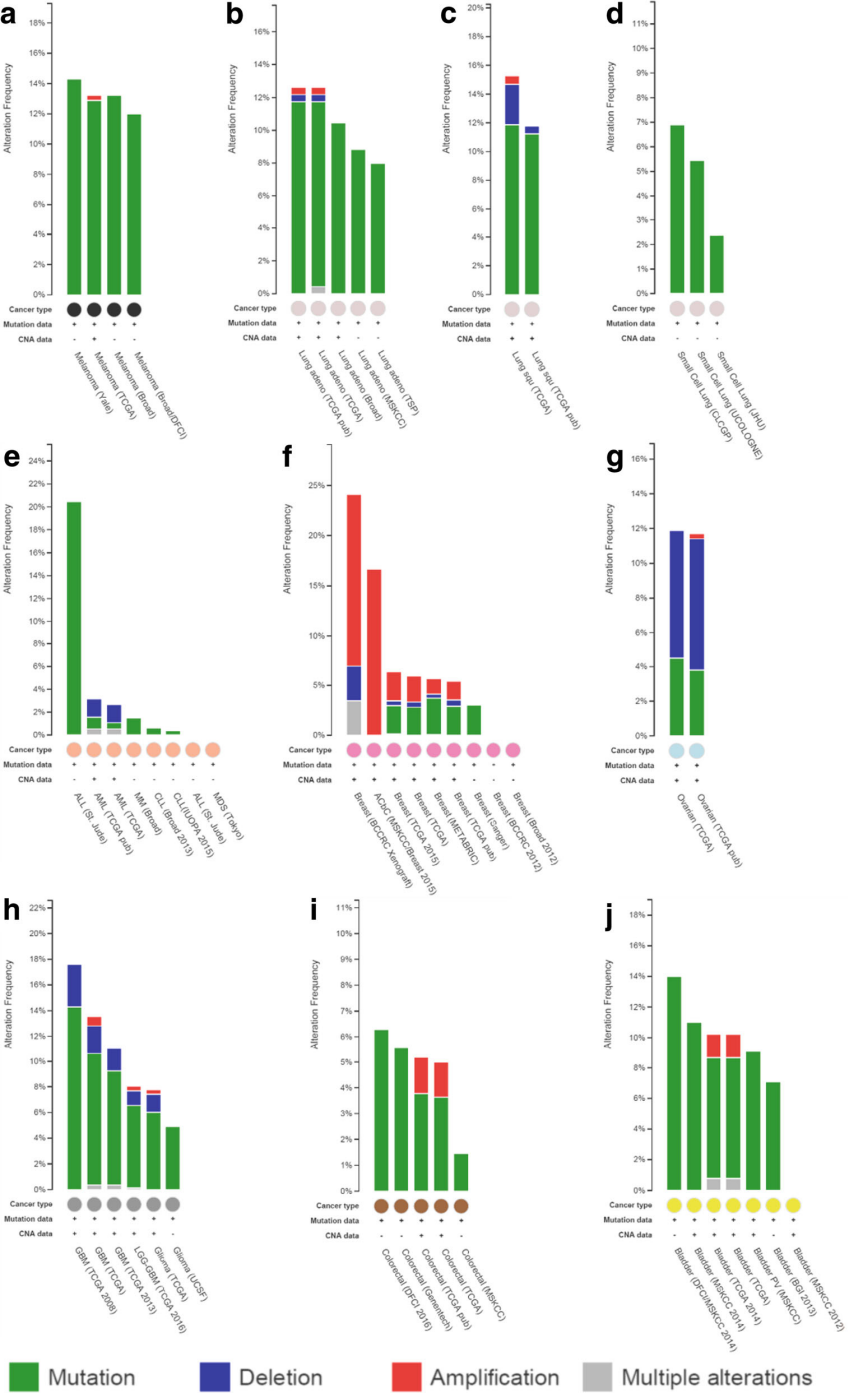
The frequency and nature of somatic *NF1* mutations in different cancer types derived from the cBio dataset. **a** Malignant melanoma. **b** Lung adenocarcinoma. **c** Lung squamous cell carcinoma. **d** Small cell lung carcinoma. **e** Acute lymphocytic leukaemia (*ALL*). Acute myeloid leukaemia (*AML*), chronic lymphocytic leukaemia (*CLL*), malignant myeloma (*MM*) and myelodysplastic syndrome (*MDS*). **f** Breast carcinoma. **g** Serous ovarian carcinoma. **h** Brain glioma, including glioblastoma multiforme (*GBM*). **i** Colorectal carcinoma. **j** Bladder transitional cell carcinoma. Mutations = single base-pair substitutions, in-frame microdeletions/insertions, frameshift microdeletions/insertions, splice site mutations (including those that can create in-frame deletions via exon skipping), nonsense mutations and frameshift insertions/deletions (shown in *green*); deletions = gross, multi-exonic and whole gene deletion identified as copy number changes (shown in *blue*); amplification = multi-exonic, whole gene duplications identified as copy number changes (shown in *red*); multiple alterations = some combination of mutations, deletions and/or amplification (shown in *grey*) [44–46]

The frequency of somatic *NF1* mutations in different sporadic tumour types derived from the literature is given in Table 1. The cBioPortal for Cancer Genomics provides a web resource for exploring, visualizing and analysing multidimensional cancer genomics data and provides graphical summaries of gene-level data from multiple platforms, shown in Fig. 1 [45].

**Table 1:** Frequency of somatic *NF1* mutations in different human neoplasms

Neoplasm	Frequency of somatic *NF1* mutations	References
Cutaneous melanoma	12–30%	[49–51, 58]
Desmoplastic melanoma	45–90%	[60, 61]
Lung adenocarcinoma	7–11.8%	[65–67, 166, 176, 177]
Lung squamous cell carcinoma	10.3–11%	[72, 177]
Acute myeloid leukaemia	3.5–23.6%	[82–85]
T cell acute lymphoblastic leukaemia	3%	[88]
Breast cancer	2.5–27.7%	[106, 177]
Ovarian carcinoma	12–34.4%	[113, 115, 170, 177–180]
Paraganglioma/phaeochromocytoma	21–26%	[121, 124, 177]
Neuroblastoma	2.2–6%	[130]
Glioblastoma	14–23%	[132, 134, 177]
Colon adenocarcinoma	3.8–6.25%	[143, 177]
Bladder transitional cell carcinoma	6–14%	[149, 167, 177]

## Main body

### Melanoma

Melanoma is a skin cancer that arises from melanocytes. Although the precise causes of melanomas are still not fully understood, environmental exposure to ultraviolet (UV) radiation from sunlight or tanning lamps certainly increases the risk of developing melanoma. Although NF1 is associated with pigmentary abnormalities such as CALMs, malignant melanoma is not a tumour type associated with NF1.

Somatic mutation analysis of melanoma by next-generation sequencing has been performed at multiple centres leading to the identification of several different pathways thought to be involved in the initiation and progression of melanoma.

The direct involvement of *NF1* in melanoma was first reported by Andersen and colleagues in 1993 who identified a homozygous *NF1* deletion in one of eight malignant melanoma cell lines which resulted in the loss of detectable *NF1* mRNA and neurofibromin protein [48]. Furthermore, the apparent absence of neurofibromin and *NF1* mRNA was recorded in a primary melanoma. This led to their proposal that *NF1* may function as a tumour suppressor gene in the development or progression of malignant melanoma. Many subsequent studies have identified additional somatic *NF1* mutations in melanoma in 12–30% of cases [45, 49–55].

RAS/MAPK pathway dysregulation has been identified as a key culprit in non-familial melanoma, leading to the discovery of *BRAF* and *NRAS* as the most commonly mutated genes [56]. Indeed, *BRAF* mutations occur in 50–70% of all cutaneous malignant melanomas, whilst *NRAS* alterations only occur in 19–28% of tumours. In both cases, these gene lesions result in constitutive activation of the MAPK pathway and are believed to be early somatic events associated with melanoma initiation [56, 57]. The high frequency of *BRAF* and *NRAS* mutations in melanomas has recently been confirmed by high-throughput next-generation sequencing (NGS) analysis which also identified additional driver mutations, including a recurrent *RAC* mutation, which is the third most frequent activating mutation in sun-exposed melanomas after *BRAF* and *NRAS* mutations [50, 51].

Inactivating *NF1* mutations have been detected in approximately 13% of melanomas, alongside mutations in other tumour suppressor genes, including *TP53*, *ARID2*, *PTEN*, *CDKN2A*, *MAP2K1* and *RB1* [51]. The impact on *NRAS* is however non-uniform, with some *NF1* mutant melanomas exhibiting full *NRAS* activation (i.e. the same activation level as oncogenic *NRAS* mutations), whereas others exhibit only partial activation [51]. In a mouse melanoma model, *NF1* mutations cooperate with *BRAF* mutations in the pathogenesis of melanoma by preventing oncogene-induced senescence, an indication that NF1 plays a key role in early melanoma development [58]. In both mouse tumour models and A375 human melanoma cell lines, Maertens and colleagues have shown that resistance to treatment was enhanced by further suppression of *NF1* by small hairpin RNA (shRNA). Furthermore, they observed that resistance to the BRAF inhibitor PLX4720 was attenuated by reconstitution of *NF1* in these cells [58]. Using RNA interference (RNAi) screening techniques, Whittaker and colleagues confirmed that *NF1* mutation is a key mechanism in BRAF inhibitor resistance. An RNAi screen, targeting more than 16,500 genes in a BRAF inhibitor-sensitive melanoma cell line, identified NF1 as the highest ranking protein affected by BRAF inhibition, and that, *NF1* knockdown abrogated the growth inhibitory effects of BRAF inhibition [53]. Indeed, it was found that *NF1* suppression led to a 31-fold increase in resistance to PLX4720, as well as a partial (7-fold) resistance to MEK inhibition, demonstrating that human melanoma samples with innate resistance to BRAF inhibition and sensitivity to a MEK inhibitor harboured *NF1* mutations [53].

Importantly, *NF1* mutations have been found in melanomas that lack both *BRAF* and *NRAS* mutations, with 25–30% of such melanomas found to harbour deleterious *NF1* mutations, thus implying that *NF1* inactivation has conferred aberrant MAPK pathway activation in these tumours [50, 51]. *BRAF/NRAS* wild-type and *NF1* mutant melanomas are strongly associated with UV damage, as evidenced clinically by the higher degree of solar elastosis and, at a molecular level, by a high proportion of C > T transitions at pyrimidine dimers and more frequent tandem CC>TT transitions [59].

A recent study based on 213 human melanoma samples identified three frequently mutated genes: *BRAF*, *NRAS* and *NF1*, with frequencies of 38.5, 28.6 and 12.2%, respectively [49]. Whilst known recurrent activating mutations were identified in *BRAF* and *NRAS*, a high number of inactivating mutations were identified in *NF1*. Notably, almost half (26/56) of *BRAF* and *NRAS* wild-type melanomas had an *NF1* mutation, most identified by loss of heterozygosity (LOH). Furthermore, *NF1* mutation-containing melanomas also harboured significantly more somatic mutations across all loci and occurred in significantly older patients, although they were associated with similar overall patient survival rates as compared to *BRAF* or *RAS* mutant or *BRAF-RAS-NF1* wild-type melanoma. In addition, all 26 *NF1* mutant *BRAF-RAS* wild-type melanomas carried mutations in other known RASopathy genes, including *RASA2*, *PTPN11*, *SOS1*, *RAF1* and *SPRED1* [49]. In contrast to Whittaker and colleagues, Krauthammer et al. also found that 6/10 *NF1* mutant cell lines were highly sensitive to a MEK inhibitor, whereas the other four were highly resistant, clearly indicating that *NF1* suppression is not always associated with either sensitivity or resistance to MEK inhibitor [49, 53].

### Desmoplastic melanoma

The highest frequency of somatic *NF1* mutations were found in desmoplastic melanomas (14/15) [60]. These melanomas are characterized by their higher propensity for local recurrence and less frequent metastatic spread to regional lymph nodes. The high frequency of *NF1* mutations in desmoplastic melanomas appears to indicate an important role for neurofibromin in the specific biology of this type of melanoma. Another recent study screened 20 desmoplastic melanomas by exome sequencing for alterations in the MAPK and PI3K signalling pathways, i.e. mutations in *CBL*, *ERBB2*, *MAP2K1*, *MAP3K1*, *BRAF*, *EGFR*, *PTPN11*, *MET*, *RAC1*, *SOS2*, *NRAS* and *PIK3C*, which were found in 15/20 (75%), with *NF1* mutations being found in 9/20 (45%) [61].

### Uveal melanoma

Melanoma of the uveal tract (i.e. iris, ciliary body and choroid) is rarer than cutaneous melanoma but is nevertheless the most common primary intraocular malignancy in adults, with inactivating mutations found in approximately 60% (23/38) of uveal melanomas [62]. Intriguingly, whilst not malignant, the Lisch nodules characteristic of NF1 is hamartomatous uveal melanocytic proliferations of the iris.

### Mucosal melanoma

Mucosal melanoma differs from cutaneous melanoma in terms of its molecular profile, with less frequent *BRAF* and more frequent *KIT* mutations but also has a poor prognosis. In a recent study [63] of a cohort of 75 tumours from patients with a mucosal melanoma, *NF1* and *RAS* mutations were identified in 18.3 and 16.9% samples, respectively, whereas 8.4 and 7% of tumour samples harboured *BRAF* and *KIT* mutations [63]. This study demonstrates that *NF1* is the most frequently occurring driver mutation in mucosal melanoma.

### Lung cancer

Lung cancer is responsible for about 10% of all cancer cases worldwide; the vast majority of which has been attributed to tobacco smoking [64]. The two main types are non-small cell lung cancer (NSCLC) in 80–90% cases and small cell lung cancer (SCLC) found in 10–15% of patients. NSCLC has multiple subtypes, including adenocarcinoma (ADC), squamous cell carcinoma (SqCC) and undifferentiated (large cell) lung carcinoma.

#### Adenocarcinoma

Approximately 40% of NSCLC are ADC, and several studies have reported somatic *NF1* mutations in some 7–11% of ADC [65–68]. The high mortality rate characteristic of this tumour type is due in part to the frequent presentation of such tumours at a locally advanced or metastatic stage and the lack of an effective advanced stage treatment [65, 69]. A number of potential novel therapeutic targets have been identified, including the activating mutations in *KRAS*, *BRAF*, *ERBB2* and *PIK3CA*; the translocations in *RET* and *ROS1*; and the loss of function or deletions of *TP53*, *NF1*, *CDKN2A* and *KEAP1* [65, 70]. Whilst *NF1* mutations were only found in 7% (13/188) of sporadic lung ADC [65], further analysis found that biallelic inactivation at the *NF1* locus may be present in as many as 23% (3/13), although it is not known whether these lesions occurred in *cis* or in *trans* [65]. Similarly, Imielinski et al. identified somatic *NF1* mutations in 10.9% (20/183) of lung ADC, of which half were found to be truncating mutations, resulting in a complete loss of function [66].

In addition to *NF1* being recurrently mutated in a subset of sporadic lung ADC patients, the MAPK pathway also appears to be an important regulatory pathway involved in tumorigenesis [65]. The TCGA research network examined the genomes, RNA and some protein from 230 previously untreated lung ADC and matched normal samples [41, 67]. In three quarters of the samples, the group identified mutations in *NF1* and other genes that activate the RTK/RAS/RAF cell signalling pathway. This study not only identified loss-of-function *NF1* defects but also demonstrated that *NF1* mutations (as well as *KEAP1* and *TP53* mutations) are far more frequent in the *BRAF-RAS* oncogene-negative subset of lung ADC. Additionally, TCGA and other groups have identified genes such as *TP53*, *KRAS*, *STK11* (*LKB1*), *EGFR* and *NF1* to be significantly mutated in ADC [67].

Markedly reduced *NF1* mRNA expression in ADC has been found to confer both an intrinsic and an acquired resistance to EGFR inhibitors [71]. By performing a genome-wide siRNA screen of both a human lung cancer cell line and a murine mutant EGFR-driven lung ADC, this revealed reduced *NF1* mRNA expression in both, and furthermore, whilst the EGFR inhibitor erlotinib failed to fully inhibit RAS-ERK signalling when neurofibromin levels were reduced, treatment of neurofibromin-deficient lung cancers with MEK inhibitor restored sensitivity to erlotinib [71].

In a recent study of 591 NSCLC, 60 had *NF1* mutations (10%) whilst 141 (24%) harboured *KRAS* mutations [68]. Approximately 25% of the *NF1* mutations co-occurred with mutations in known oncogenes: *BRAF*, *ERBB2*, *KRAS*, *HRAS* and *NRAS*. Therapeutic strategies targeting *KRAS* activation, including the use of inhibitors of MAP kinase signalling, may warrant investigation in *NF1* mutant tumours. Additional tumour suppressor inactivation pattern studies may help to inform novel treatment strategies.

#### Squamous cell carcinoma

According to the TCGA, somatic *NF1* changes are present in approximately 12% of squamous cell lung cancers (SqCC), of which four distinct subtypes have been identified: classical, primitive, basal and secretory expression [72]. The basal expression subtype was found to harbour *NF1* alterations, suggesting a potential direction for the treatment of such tumours. The information from the TCGA studies has highlighted the involvement of *NF1* in both lung ADC and SqCC and served to improve our understanding of the genetic pathways that lead to lung cancer [72].

Transcriptome analysis of 153 tumour samples, including ADC, SqCC, large cell lung cancer, adenoid cystic carcinomas and derived cell lines, has been integrated with the data from The Cancer Genome Atlas and other published sources [73]. This confirms the previously reported *CD74-NRG1* fusion and also suggests that the *NRG1*, *NF1* and Hippo pathway fusions may play important roles in tumours without known driver mutations and that this prognostic factor may be associated with poor survival [73]. Several different gene fusions, viz. *NF1-GOSR1*, *NF1-PSMD11*, *NF1-NLK*, *NF1-DRG2* and *NF1-MYO15A*, were also detected by transcriptome sequencing of lung cancers [73]. Interestingly, both lung ADC and SqCC DNA displayed a significantly increased frequency of guanine (cytosine) to thymine (adenine) mutations, a type of mutation associated with exposure to tobacco smoke [68]. Lung ADC genomes also manifest regional heterogeneity in terms of the distribution of mutations with sequencing data from lung cancer studies clearly indicating that lung cancer, at the molecular level, is a highly heterogeneous disease. Indeed, the mutational landscape of lung ADC is substantially different from that of SqCC of the lung or SCLC [74], with frequent receptor tyrosine kinase mutations found in lung ADC, that are rarely encountered in either SqCC or SCLC [75].

Mutations in *TP53*, *KRAS*, *LKB1*, *NF1* and *RBM10* are enriched in transversion-high tumours, whilst mutations in *EGFR*, *RB1* and *PIK3CA* and in-frame insertions in the receptor tyrosine kinases *EGFR* and *ERBB2* are enriched in transversion-low tumours [75]. The transversion-high group was found to be strongly associated with past or present smoking (*P* < 2.2 × 10^−16^) [72, 74, 75].

To compare lung ADC and SqCC and to identify new drivers of lung carcinogenesis, Campbell and colleagues examined the exome sequences and copy number profiles of 660 lung ADC and 484 lung SqCC tumour normal pairs [74]. They observed median somatic mutation rates of 8.7 mutations/Mbp and 9.7 mutations/Mbp for lung ADC and SqCC, respectively. At least 38 genes were significantly mutated in lung ADC and 20 genes in SqCC; however, only six genes, *TP53*, *RB1*, *ARIDIA*, *CDKN2A*, *PIK3CA* and *NF1*, were significantly mutated in both tumour types, and of these, *TP53*, *CDKN2A* and *PIK3CA* mutations had a significantly higher frequency in lung SqCC. Recurrent alterations in lung SqCC were more similar to those of other squamous carcinomas than to alterations in lung ADCs, whilst the significantly mutated genes in lung ADC were most similar to those associated with glioblastoma and colorectal cancer.

#### Small cell lung cancer

Although there is a paucity of data for small cell lung cancer (SCLC), the frequency of *NF1* mutations in SCLC was found to be 2.4 and 6.9% in two independent studies [76, 77]. In a subsequent study of 98 SCLC, DNA was sequenced to a high, uniform coverage and analysed for all classes of genomic alterations [78]. Of the seven most commonly altered genes identified, only one (*RICTOR*) was considered to be actionable in terms of treatment. The most common non-actionable genomic alterations were found in *TP53* (86% of SCLC cases), *RB1* (54%) and *MLL2* (17%), with *NF1* mutations identified in only 3% of SCLC, consistent with the earlier studies.

### Myeloid malignancies

Myeloid malignancies are clonal disorders characterized by acquired somatic mutations in various haematopoietic progenitors. Constitutional *NF1* mutations are known to predispose individuals to myeloid malignancies such as chronic myelomonocytic leukaemia (CMML), JMML and acute myeloid leukaemia (AML) [79]. Somatic 17q11 deletions encompassing *NF1* have been described in many adult myeloid malignancies [80]. More generally, the RAS signalling pathway has been found to be fundamental in the development of myeloid malignancies, with somatic activating mutations in *NRAS* and *KRAS* genes estimated to be present in 20 to 40% of diagnosed cases of AML, CMML and JMML [81]. Recent advances in understanding the genetic basis of myeloid malignancies have provided important insights into the pathogenesis of AML. Whilst somatic *KRAS* and *NRAS* mutations are frequently found in AML, mutations in other RAS signalling pathway genes, including *NF1*, occur at lower frequencies, although the reported frequency for *NF1* somatic mutations ranges quite widely from 3.5 to 23.6% [79, 82–85].

Parkin and colleagues identified *NF1* mutations in 7% of cases with AML, with a further 12% displaying copy number alterations (CNAs) involving *NF1*, mainly heterozygous deletions [85]. The absence of *NF1* expression was observed in 7% of adult AML associated with an increased RAS-GTP level. In another study of AML with CBHB-MYHII rearrangements, 16% of the samples harboured *NF1* deletions [84]. However, high-resolution studies have failed to provide any evidence for frequent *NF1* alterations in de novo AML, although they suggested that *NF1* mutations may contribute to tumour progression [82]. In this study, the authors screened a total of 488 previously untreated de novo AML patients for the *NF1* deletion using either array comparative genomic hybridization (aCGH) or real-time quantitative PCR/fluorescence in situ hybridization approaches. Using aCGH, a small ~0.3 Mbp minimally deleted region involving *NF1* was defined; the overall frequency of *NF1* deletion was 3.5% (17/485). Furthermore, *NF1* deletion was significantly associated with abnormal cytogenetics and a monosomal karyotype, whilst only one of five *NF1*-deleted patients acquired a coding mutation in the remaining allele. This study indicates that *NF1* microlesions are infrequent in de novo AML and may be secondary events in leukemic progression.

#### Myelodysplastic syndrome

The frequency of *NF1* changes in myelodysplastic syndrome has been found to vary between 0 and 9% [86, 87].

#### T cell acute lymphoblastic leukaemia

T cell acute lymphoblastic leukaemia (T-ALL) is a variant of acute lymphoblastic leukaemia (ALL), with features similar to some types of lymphoma. It accounts for about 15 and 25% of ALL in paediatric and adult cohorts, respectively.

T-ALL is a highly aggressive malignancy, characterized by rapid progression and high relapse rates [79]. Somatic mutations in a number of established T-ALL drivers such as *KRAS*, *NRAS*, *PIK3CA*, *PTEN*, *NOTCH1*, *PHF6* and *NF1* have been identified in T-ALL cell lines and patient samples [88]. Somatic *NF1* mutations have been found in 27.3% (9/33) of the T-ALL cohort; however, only 12.1% (4/33) were non-synonymous mutations [88]. The type 1 *NF1* microdeletion (1.4 Mb) was reported in 2.9% (3/103) of T-ALL patients [79]. None of these three individuals with the microdeletion exhibited any clinical characteristics of NF1.

#### Juvenile myelomonocytic leukaemia

JMML is a myeloproliferative neoplasm (MPN) of childhood, occurring when too many immature white blood cells (myelocytes and monocytes) are made in the bone marrow. In 1997, Side and colleagues reported constitutional *NF1* mutations in 15% of JMML patients [89]. JMML generally carries a very poor prognosis, with the only curative treatment being haematopoietic stem cell transplantation.

JMML was once considered a unique example of RAS-driven oncogenesis because it was thought to be initiated by mutually exclusive mutations in the RAS genes (*NRAS* or *KRAS*) or in several RAS pathway regulators (*PTPN11*, *NF1* or *CBL*) [90].

In an exploration of the somatic mutation landscape of 30 patients with syndromic (*n* = 8) or sporadic (*n* = 22) JMML, a combination of genome-wide DNA array analysis, whole-exome sequencing and targeted sequencing was used in paired germline and tumour samples [90]. In total, 85 somatically acquired genetic alterations were found in 83% (25/30) of patients in this sub-cohort. Genes containing somatic variants detected by whole-exome sequencing, or previously reported to be mutated in JMML, were then sequenced in the full cohort of 118 JMML cases. A total of 122 secondary clonal abnormalities, in addition to initiating RAS pathway mutations, were identified in 49% (58/118) of patients [90]. In addition, sequencing of isolated myeloid colonies demonstrated the coexistence of multiple RAS hits in the same myeloid progenitors in three of the JMML cases tested, challenging the concept of mutually exclusive RAS pathway mutations.

The polycomb recessive complex 2 (PRC2) is involved in cellular differentiation, maintenance of cell identity and proliferation as well as stem cell plasticity [91] and also drives myeloid malignancies. *Nf1*/*Kras* double-mutant mice have been shown to develop myeloid malignancies with reduced latency and increased severity in comparison to mice with only one of the two defects because copy number variations (CNVs) in *Nf1/Kras* mutant mice frequently resulted in haploinsufficiency for PRC2 core subunits (*SUZ12* or *EZH2*) or PRC2-associated factors necessary for optimal PRC2 activity (*AEBP2*, *CDYL* or *JARID2*) [92]. In addition, haploinsufficiency for multiple genes that regulate PRC2 function can cooperate in myeloid transformation, and other mutations in JMML target a small number of pathways specifically, including components of the RAS and PRC2 networks [93, 94]. Thus, RAS activation is a major player, and other pathways such as PRC2 are also important. Notably, PRC2 also plays a role in the development of MPNSTs. Loss of function of PRC2 (due to mutations in *EED* or *SUZ12*) is also found in the vast majority of sporadic, NF1-associated, and radiotherapy-associated MPNSTs (where PRC2 loss amplifies Ras-driven transcription) [95, 96].

In a recent study focussing on characterization of serial samples from JMML patients at diagnosis and then beyond through relapse and transformation to AML, mutations were found in *NF1*, *NRAS*, *KRAS*, *PTPN11* or *CBL* in 85% of patients, as well as recurrent mutations in other genes involved in signal transduction, splicing, PRC2 and transcription. The number of somatic alterations present at diagnosis appeared to be important for the outcome of JMML [97].

### Breast cancer

The *NF1* gene is reported to be frequently mutated in sporadic breast cancers, although in only a few studies has mutation frequency been published. NF1 patients have an increased risk of developing breast cancer as compared to the general population [8, 98]. In particular, women under the age of 50 with NF1 have an increased (4–5-fold) risk of developing breast cancer (standardized incidence ratio for women under 50) and also a 3.5-fold increased fatality risk (proportionate mortality ratio) [98–100]. A predisposition to breast cancer in NF1 patients has led researchers to postulate the potential involvement of somatic *NF1* mutations in initiating and driving the malignant transformation and progression of sporadic breast cancer. A number of breast cancer genome sequencing studies have identified *NF1* as one of a number of novel, recurrently mutated genes in sporadic tumours which could potentially be targeted in a therapeutic context [101, 102].

It was Ogata and colleagues, working with established breast cancer cell lines in 2001, who first identified a role for *NF1* in the malignant transformation of mammary cells [103]. Further analysis of the *NF1* deletion-bearing tumours revealed significantly higher levels of active RAS, indicating that RAS signal transduction pathway dysregulation, through *NF1* loss, may be responsible for driving malignancy in these cells. Neurofibromin was found to be below detectable levels in the highly malignant and treatment-resistant MB-231 breast cancer cell line as compared with four other less aggressive cell lines. Additionally, the MB-231 cells exhibited a 10-fold increase in pMAPK levels as a result of activated Ras, despite there being no changes in p120^GAP^. Hence, this study suggested that under-expression of *NF1* and reduced neurofibromin activity may have a direct influence on malignant transformation and resistance to anti-cancer agents [103]. This is consistent with other studies and goes some way towards accounting for the presence of the somatic *NF1* mutations found in sporadic breast tumours.

The mouse model *Chaos3* is characterized by the spontaneous development of mammary tumours, due to a mutation in *Mcm4* leading to chromosomal instability through disruption of the MCM2-7 complex [104, 105]. Somatic *NF1* deletions were found in almost all (59/60) of the mammary tumours studied in this mouse model, and upon subsequent examination of TCGA data, it was noted that *NF1* is somatically mutated or deleted in 27.7% of human breast cancers [105, 106].

Large-scale NGS to compare primary and recurrent breast cancer has found mutations in recurrent tumours which were not present in matched primary tissue [107]. However, the difficulties inherent in studying recurrent tumours mean that the sample size was necessarily small in this study, with only 74 matched tumours from 43 patients across the various breast cancer subtypes. So, the precise role for *NF1* in breast cancer is still unclear and further studies are required.

CNAs dominate the breast cancer genome, with *NF1* gene amplification being a particular feature not seen in the other tumour types in which *NF1* mutations are observed (Fig. 1), suggesting that gain of neurofibromin function is especially important in breast cancer biology. In contrast, genes generally mutated in breast cancers are subject to a low frequency of somatic mutations, including single nucleotide mutations and indels in driver genes [105, 106].

Large-scale efforts by the TCGA and ICGC have contributed greatly towards determining the identity of genes mutated in breast cancer, but analysis of clinical associations in these data sets is limited by the scarcity of long-term patient follow-up data and the stringent criteria used for sample selection (e.g. tumour size, malignant cellularity) [41, 42].

In a recent study based on 2433 molecular profiles of breast cancer, it was noted that high levels of intra-tumour heterogeneity was generally associated with a worse clinical outcome, with one exception: highly aggressive breast tumours with 11q13–14 amplification had low levels of intra-tumour heterogeneity [108, 109]. Inactivating *NF1* mutations were also found to be associated with breast cancer severity score in oestrogen receptor-negative tumours.

As with melanoma and neuroblastoma, inactivation of *NF1* in breast cancer is associated with resistance to drug therapy. A potential mechanism for *NF1* and drug resistance in breast adenocarcinoma has been suggested following analysis of the MCF-7 breast cancer cell line [110]. Silencing of *NF1*, amongst several other genes, has been shown to confer a tamoxifen-resistant phenotype, although it was noted that resistance- or sensitivity-specific gene expression patterns may give a better prediction of treatment outcome as compared to single genes [111]. This is potentially of great clinical importance, of course, as, although tamoxifen is one of the most widely used anti-breast cancer agents, it is now apparent that up to ~40% of early-stage breast cancer patients who receive tamoxifen as an adjuvant therapy will ultimately develop tamoxifen resistance and relapse [111, 112].

### Ovarian cancer

High-grade serous ovarian carcinoma (HGOSC) is the most common and malignant form of ovarian tumours accounting for up to 70% of all ovarian cancer cases. Some serous cancers may initiate in cells at the distal end of the fallopian tube, then spread to the ovary. There are different subtypes of epithelial ovarian cancer including mucinous, endometrioid, clear cell, undifferentiated or unclassifiable; therefore, HGOSC is a molecularly and clinically heterogeneous disease which accounts for the majority of ovarian cancer deaths.

More than a third of all ovarian serous carcinomas (OSCs) harbour somatic *NF1* mutations, identifying an alternative target for treatment and an additional prognostic marker. This is of particular importance when considering the disease heterogeneity, high relapse and fatality rates [113, 114].

A role for *NF1* in ovarian serous carcinoma (OSC) was first proposed by Sangha et al. in 2008 [113]. Genome-wide microarray analysis of 36 primary OSC identified homozygous *NF1* deletions in two tumours. This group subsequently screened 18 ovarian carcinoma-derived cell lines and 41 primary OSC for additional *NF1* alterations, with 8/18 cell lines exhibiting marked reduction or no expression of *NF1*. Homozygous *NF1* gene deletions and *NF1* splicing mutations were identified in 9/41 primary OSC. Additionally, tumours and cell lines with *NF1* lesions were found to lack *KRAS* and *BRAF* mutations, whilst exhibiting Ras pathway activation [113].

The Cancer Genome Atlas project analysed the expression of mRNA and microRNA, promoter methylation and DNA copy number in 489 HGOSC and performed genomic DNA analysis in 316 tumours. Loss of *NF1* function was identified in 12% (37/316) of samples, and of these, 24 had deletions, one had a duplication and the remaining (12) samples harboured other somatic mutations. The Australian Ovarian Cancer Study (AOCS) specifically examined CNAs and reported regions of copy number loss at the *NF1* locus in 34% (137/398) of ovarian cancer samples, comprising 157 serous adenocarcinomas from the TCGA cohort and a further 241 samples, of both endometrioid and serous subtypes [115].

HGOSC shows a simple mutational profile, with *TP53* nearly always mutated, but with other genes, including *NF1*, mutated at a low frequency [116]. Approximately 50% of all HGSOCs exhibit homologous recombination (HR) deficiency, with such tumours being highly sensitive to PARP inhibitors [117]. However, *NF1* mutations identified in advanced HGOSC are associated with resistance to treatment because of the acquisition of different new mutations within the gene [116–118].

### Paragangliomas and phaeochromocytomas

Phaeochromocytomas are rare tumours (annual incidence of 1–6 million per year) that develop from neural crest-derived chromaffin cells and produce excess catecholamine, resulting in hypertension and flushing. Despite being rare in the general population, the frequency of occurrence amongst NF1 patients is much higher, with 0.1–6% developing a phaeochromocytoma [119, 120].

*NF1* is one of a number of known paraganglioma and phaeochromocytoma susceptibility genes, constitutional mutations in which are responsible for inherited tumour syndromes. Somatic *NF1* mutations occurred in 35/161 (21.7%) of sporadic phaeochromocytomas, with the majority exhibiting LOH and low *NF1* mRNA expression [121–123], whilst somatic mutations in the susceptibility genes *NF1*, *MAX*, *RET*, *VHL*, *SDHA*, *SDHB*, *SDHC*, *SDHD*, *SDHAF2*, *KIF1Bβ* and *TMEM127* are present in 11–19% of sporadic cases [124–126]. It has also been demonstrated that the majority (83%, 35/42) of sporadic phaeochromocytomas harbour a CNA in at least one of these susceptibility genes, thereby altering respective protein expression levels [123]. This is in addition to the 26% (11/42) of sporadic paragangliomas and phaeochromocytomas that have lost one *NF1* allele, associated with a reduction in *NF1* mRNA level. Furthermore, 10 of 11 tumours were also observed to harbour a somatic protein-truncating *NF1* mutation in the second allele [121]. This study also identified a correlation between *NF1* mutations and a biochemical phenotype: paragangliomas and phaeochromocytomas harbouring a somatic *NF1* mutation were found to display higher plasma levels of normetanephrine (*P* = 0.005) and metanephrine (*P* = 0.0025), markers for catecholamine-secreting tumours [121]. This could be of significance as plasma catecholamine levels are used in the diagnosis of phaeochromocytoma and paraganglioma; however, these findings were reported in only a small sample group and the biochemical data was non-centralized and incomplete, limiting their overall significance [123].

A large-scale analysis of a cohort of 202 paragangliomas and phaeochromocytomas, collected by the *Cortico et Médullosurrénale: les Tumeurs Endocrines* (COMETE) network, examined CNAs, somatic and constitutional mutations in known susceptibility genes [124]. Almost a quarter (25/119) of the sporadic phaeochromocytomas/paragangliomas carried an inactivating *NF1* mutation, of which 21/25 were associated with the loss of the wild-type allele. Of all the somatic mutations identified in the study, 56% were located in *NF1*, showing that *NF1* is frequently mutated in phaeochromocytomas/paragangliomas [124].

### Neuroblastoma

Neuroblastoma is a neuroendocrine tumour that originates from neural crest cells of the sympathetic nervous system, with most tumours developing in the abdomen. Neuroblastoma is the second most common solid tumour in childhood and accounts for 8% of all childhood cancers. The treatment for neuroblastoma includes surgery, chemotherapy, radiation and bone marrow transplantation. Familial neuroblastoma cases comprise only a small fraction (~1–2%) of all neuroblastoma cases, and their genetic aetiology is relatively well understood [127, 128]. In contrast, far less is known of the genetic aetiology of sporadic neuroblastomas, despite their accounting for the majority of cases.

It was a quarter of a century ago when *NF1* was first reported to play a role in the development of neuroblastoma. In this study, 4/10 neuroblastoma cell lines were observed to express either a reduced level or a complete absence of neurofibromin, with *NF1* mutations being identified in two of these cell lines [129]. Furthermore, it was demonstrated that the introduction of a normal human chromosome 17 into a neuroblastoma cell line suppressed its tumorigenicity. Several *NF1-*deficient neuroblastoma cell lines exhibited only moderately elevated *Ras*−GTP levels, in contrast to *NF1* tumour cells, indicating that neurofibromin can contribute differently to the negative regulation of RAS in different cell types [130, 131].

Somatic *NF1* mutations in neuroblastomas have been correlated with reduced expression of neurofibromin and poor patient prognosis, whilst higher levels of expression are associated with longer progression-free survival [130, 131]. Hölzel and colleagues also reported a loss of neurofibromin expression in 8/25 neuroblastoma cell lines and that a further SNP analysis of 20 neuroblastoma cell lines detected 50% (10/20) with abnormal *NF1* alleles [130]. Genomic aberrations in *NF1* were also found in primary neuroblastomas but at a lower frequency of 6% (5/83).

A large-scale RNAi screen revealed an association of *NF1* loss in neuroblastoma cell lines with resistance to retinoic acid (RA) treatment which is used as targeted therapy in the treatment of neuroblastomas. Loss of *NF1* activates RAS-MEK signalling, which in turn represses *ZNF423*, a critical transcriptional coactivator of the retinoic acid receptors; neuroblastomas with low levels of both *NF1* and *ZNF423* have an extremely poor outcome. However, inhibition of MEK signalling downstream of *NF1* restores responsiveness to RA, suggesting a potential therapeutic strategy to overcome RA resistance in *NF1*-deficient neuroblastomas [130].

### Glioblastoma

Glioblastomas are tumours that arise from astrocytes that comprise the supportive tissue of the brain. These tumours are usually aggressive as the cells divide rapidly and are also supported by a large network of blood vessels. The most aggressive subtype is glioblastoma multiforme (GBM) which is the most frequent form of brain cancer in adults, renowned for its lethality and poor prognosis and is thus an important target of study [132, 133].

Glioblastoma-associated *NF1* somatic mutations are well described [132, 134, 135], with recurrent driver mutations being identified in *NF1* and a number of other candidate genes (*IDH1*, *TP53*, *CDK4*, *EGFR*, *PI3KR1*, *PIK3CA*, *PTEN*, *RB1* and *CDNK2A*) in GBM [132]. *NF1* mutations were identified in at least 15% (16/105) of all GBM by Parsons and colleagues, although chromosomal translocations or epigenetic changes were not tested in this cohort [132].

A TCGA analysis assessed levels of gene expression, CNAs and DNA methylation in a cohort of 206 glioblastoma tumour samples, with recurrent mutations in *NF1*, *AKT3*, *PRK3R1* and *PARK2* being identified, and with 14% (13/91) of samples found to contain at least one somatic *NF1* mutation. Verhaak and colleagues subsequently performed large-scale genomic analysis of these TCGA data, dividing glioblastoma cases into four subtypes: proneural, neural, classical and mesenchymal [136]. They found that GBM with *NF1* and *PTEN* alterations had a distinct mesenchymal-like expression profile, with 53% of mesenchymal cases having an *NF1* mutation. The mutual exclusivity of *NF1* and *BRAF* mutations in GBM has also been reported [134].

In animal models, inactivation of *TP53* and *PTEN* may cooperate with *NF1* loss in the development of glioblastoma [137]. Haploinsufficiency of *NF1* is also reported to increase astrocyte proliferation and enhancement of angiogenesis in *Nf1* ^*+/−*^ heterozygous mouse models [138, 139].

### Colorectal cancer

Colorectal cancer (CRC) is one of the leading causes of cancer-related deaths in the western world, with at least 50% of CRCs exhibiting dysregulation of the RAS/MAPK pathway. Reports of the type of *NF1* mutations in CRC vary widely, with *NF1* LOH first reported in 14–57%, and reported gains in part of, or even a complete duplication of, the *NF1* gene in 17% of CRC [140–142]. The 2012 TCGA genome-scale analysis of 212 CRC found that 24 genes were predominantly mutated, including *NF1* in approximately 5.6% (11/212) of cases [143]. Subsequent studies have confirmed this, with *NF1* mutations being identified in 5.6% (4/72) and 5.8% of cases (39/619), respectively [144, 145].

Several critical genes and pathways, such as WNT, RAS/MAPK, PI3K, TGF-β, P53 and DNA mismatch repair, are recognized in the initiation and progression of CRC [146, 147]. Although genetic alterations in the PI3K and RAS/MAPK pathways are common in CRC and *NF1* alterations have been detected in 5–6% of cases, it remains unclear as to whether *NF1* mutations in CRC are related to chemotherapeutic effect.

### Urinary tract transitional cell carcinoma

The best documented molecular factors involved in urothelial transitional cell carcinoma (TCC) are the *RAS* proto-oncogene activation and *TP53* mutations. Alterations in *NF1* gene expression in TCC were first reported in 1999 [148], where decreased *NF1* gene expression was observed in 83% (23/29) of TCC specimens (as estimated by immunohistochemistry), whilst *NF1* mRNA levels were markedly lower in TCC tissue as compared with those in adjacent non-neoplastic urothelium. Neurofibromin levels were also decreased in high-grade TCC, suggesting that alterations of *NF1* gene expression might be involved in urinary TCC carcinogenesis. Whole genomic analysis performed on 35 stage IV urothelial cancers that had relapsed and progressed after primary surgery and conventional chemotherapy revealed *NF1* mutations in two cases (6%) [149]. Integrated analysis of 131 urothelial carcinomas showed recurrent mutations in 32 genes, with 14% of tumours having *NF1* mutations.

### Other malignant tumours

There are a number of other malignant tumour types that have been found to harbour *NF1* alterations including neuroendocrine prostate cancer (24%), myxofibrosarcomas (10.5%) and pleomorphic liposarcomas (8%), pancreatic cancer (11%), gastric adenocarcinoma (10%) and rhabdomyosarcoma (7%) [44, 45]. Somatic *NF1* mutations have also been detected in 41–72% of sporadic MPNSTs, showing that *NF1* inactivation plays a major role in the development of this tumour type [96].

### General discussion

Neurofibromatosis type 1, caused by constitutional inactivating mutations in the tumour suppressor gene *NF1*, is a neurodegenerative disorder predisposing individuals to both benign and malignant tumours [150–152]. Additionally, somatic mutations of *NF1* are also frequent in desmoplastic, cutaneous and mucosal melanoma, high-grade serous ovarian cancer, breast cancer, phaeochromocytomas and paragangliomas, glioblastoma multiforme, myeloid malignancies, neuroblastoma, and colorectal and urinary bladder transitional cell carcinoma (Table 1). Aberrations in neurofibromin result in the dysregulation of the RAS/MAPK pathway leading to unregulated cell growth and proliferation. The related mTOR pathway and other downstream activators and effectors of RAS including PI3K are also involved in cancer [17, 153].

Mutations (chromosomal aberrations, nucleotide substitutions and epigenetic aberrations) in a subset of candidate genes are likely to confer a growth advantage resulting in the development of cancer. Cancer encompasses more than 100 different diseases, the study of which provides insight into both the commonalities and differences between and amongst various types and subtypes of cancer [147]. In order to understand this complex disease and to develop novel targeted therapeutics, it is essential to characterize the somatic mutational spectra in each cancer genome in order to facilitate our understanding of the biological processes underlying the cancer as well as the pathways of evolutionary progression. The availability of the human genome reference sequence enabled the rapid resequencing of cancer genomes, leading to the discovery of many additional cancer genes, revealing for the first time the molecular heterogeneity of cancer genomes and identifying therapeutic targets. To improve the diagnosis and treatment of cancer patients, several large-scale cancer genomics projects, e.g. the TCGA, ICGC, cBioPortal and COSMIC, have been undertaken in recent years [41–44]. These pan-cancer projects have generated high-throughput data which provide valuable opportunities to understand the biology, initiation and progression of human cancers. One caveat, however, is distinguishing artefactual DNA damage from the bona fide mutations that actually occurred in the tumour, given that it has been reported that mutagenic damage accounts for the majority of the erroneous identification of variants with low to moderate (1 to 5%) frequency in whole (cancer) genome sequencing studies [154].

Generally, a large number of mutations occur in cancer genomes, such as somatic mutations, CNAs, methylation aberrations and histone modifications. It is critical to distinguish driver mutations and driver genes (which contribute to the progression of cancer from normal to malignant states) from passenger mutations and passenger genes (which accumulate in cells but do not contribute to cancer development). There is a subtle difference between a driver gene and a driver gene mutation. A driver gene harbours driver gene mutations but may also harbour passenger gene mutations. A driver mutation typically confers upon a tumour only a very small growth advantage, which may be as low as a 0.4% increase in the difference between cell birth and death rates [155]. More recently, Bozic and colleagues have shown that the first, and hence most abundant, passenger mutations are influenced both by the mutation rate and by the death-birth ratio of the cancer cells [156]. It should be appreciated that whilst passenger mutations do not, by definition, exert a strong selective growth advantage, they are not entirely neutral. Indeed, many are deleterious in terms of their effect on cellular proliferation and cancer progression [157, 158]. It should also be appreciated that whilst the damaging effect of a non-synonymous passenger mutation is on average 100 times smaller than the effect of a driver mutation, passengers are 100 times more numerous than drivers [158]. The paucity of drivers in a sea of passenger mutations represents a challenge to identifying the former. This task is made all the more daunting by the possibility that drivers and passengers are not discrete entities but rather lie along a continuum which includes latent driver mutations which ‘behave as passengers but coupled with other emerging mutations, drive cancer development and drug resistance’ [159].

In 2004, Futreal and colleagues published a ‘Census of human cancer genes’ which aimed to list all genes that are causally implicated in tumorigenesis. This census has been kept up to date and currently includes 602 entries [43, 160]. This implies that more than 2% of all human genes are implicated in cancer. Of these, approximately 90% have somatic mutations in cancer; 20% have germline mutations that predispose to cancer; and 10% harbour both somatic and germline mutations. A second resource, the Network of Cancer Genes (NCG) contains a total of 1053 ‘cancer genes’ whose possible involvement in cancer has been inferred by statistical means [161]. The number of genes recognized as being cancer-associated is likely to increase as new techniques are devised to identify the function of the associated proteins [162, 163].

### Cooperativity and exclusivity of *NF1* somatic mutations

Mitogen-activated protein kinases (MAPK) and phosphoinositide-3 kinase (PI3K) pathways are key cellular growth regulators. In a normal cell, these control cell growth and survival but are often disrupted in a malignant cell with a deregulated MAPK or PI3K pathway. It is now well recognized that the focus should be upon cellular pathways rather than on individual genes to achieve a full understanding of cancer biology. Therefore, defining driver pathways is an important step to understanding the molecular mechanisms underlying cancer. Previous studies have focussed mainly on identifying the alterations in cancer genomes at the individual gene or single pathway level. However, a great deal of evidence indicates that multiple pathways often function cooperatively in carcinogenesis and other key biological processes. A common and restricted number of driver genes and pathways are probably responsible for most common forms of cancer [40, 147].

In general, mutations of the genes in one pathway usually exhibit mutual exclusivity, because a single mutation is usually enough to disturb one pathway and any further hits in other components of that pathway confer no added selectable advantage. Thus, sporadic tumours with *NF1* mutations are mutually exclusive for mutations in MAPK kinase 1 (*MAP2K1*) or *NRAS*. Strongly activating ‘canonical’ mutations in oncogenes (for example G12D or G12V mutations in KRAS) can drive cancer formation on their own and are known to be epistatic in relation to other canonical mutations within the same pathway [164]. However, whether there are, for example, ‘non-canonical’ mutations that weakly activate oncogenes or only partially inactivate tumour suppressor activity and yet can drive cancer formation is less clear. Examination of genomic data from the Cancer Cell Line Encyclopedia (CCLE) and TCGA has indicated that whilst canonical *KRAS* mutations do not occur with increased frequency in the context of *NF1* mutations, non-canonical *KRAS* mutations certainly do, suggesting that such pairs of mutations might act together to confer a selective advantage in human tumours [164]. Activation of RAS guanine nucleotide exchange factors (RAS-GEFs) was predicted to have similar effects to neurofibromin loss and that non-canonical *KRAS* mutations co-occur with RAS-GEF mutations in TCGA and CCLE data [164]. Furthermore, increased frequencies of mutations in both *NF1* and other RAS pathway activators or effectors have been found which suggests that this principle could apply more broadly to other genes in the RAS network and possibly to other oncogenic signalling pathways [58]. Subsequently, *NF1* loss has been described as a key mediator of acquired and intrinsic *BRAF* inhibitor resistance following a high-throughput short hairpin RNA screening approach [53]. Furthermore, on the basis of analyses of somatic co-mutation patterns in the TCGA data sets (cBio Portal for Cancer Genomics), 9.6% of melanomas with *NF1* mutations also have mutations in *BRAF*, *NRAS* or *RAF1* [47]. But, whilst mutant *NF1* is known to cooperate with RASopathy genes (*RASA2*, *PTPN11*, *SOS1*, *RAF1* and *SPRED1*) in melanoma and although *NF1* is found to be frequently mutated (25–30%) in melanomas harbouring wild-type *BRAF* and *NRAS*, it is curious that melanoma is not a tumour type associated with NF1 [8, 49–51].

The capacity of *NF1* mutations to act both cooperatively and exclusively without *BRAF* and *NRAS* mutations in melanoma may be mediated through pathways other than the MAPK pathway. Maertens and colleagues have identified increased activation of the PI3K/AKT/mTOR pathway in *BRAF*/*NF1* double mutants, and a combinatorial MEK marker and mTOR inhibitor treatment has proven effective in many MEK inhibitor-resistant neoplasms [58]. In a glioblastoma animal model, *NF1* cooperates with both *TP53* and *PTEN*, but no co-occurrence of *NF1* and *BRAF* mutations is seen [137]. Moreover, whilst simultaneous inactivation of *Nf1* and expression of K-Ras^G12D^ in mouse haematopoietic cells results in AML that was fatal in primary mice within 4 weeks, in ovarian serous carcinomas, cooperation between mutant *TP53* and *NF1* results in a poor prognosis [92, 117]. In addition, an association between inactivated *NF1* and *ZNF423* levels in neuroblastomas has been identified as a putative prognostic marker [130].

It should be appreciated that the same gene can function in completely opposite ways in different cell types. In melanomas harbouring *BRAF* V600E mutations, a BRAF inhibitor induces remission of the tumour; however, the same drug is ineffective in colorectal cancer cells harbouring identical mutations. This has been attributed to the expression of EGFR which occurs in some colorectal cancers, but not in melanomas [165].

Despite all the cancer genome information available regarding *NF1*, it remains unclear why NF1 patients are predisposed only to certain types of tumours. Why, for example, are NF1 patients not predisposed to lung tumours given that at least 10% of all sporadic lung cancers have *NF1* mutations [8, 65, 72, 166]?

### NF1 and drug resistance

The RAS/MAPK pathway, with an important role in cancer biology, is a prime target for anti-cancer agents; however, the presence of an *NF1* mutation, resulting in reduced expression of neurofibromin, confers resistance to several therapeutic drugs. Furthermore, *NF1*-associated drug resistance to RAF and EGFR inhibitors, tamoxifen and retinoic acid, has been observed in melanoma, lung cancers, breast cancers and neuroblastoma, respectively, and melanoma cells with *BRAF*/*NF1* mutations develop resistance to BRAF inhibitors [58, 111, 130, 167]. It is not clear whether the specific nature of the mutations could have exerted an influence on the sensitivity of the drug, as complete inactivation of *NF1* has been noted to confer sensitivity to rapamycin in AML [85].

### Mutational spectrum

Large constitutional *NF1* deletions, encompassing the *NF1* gene and many adjacent genes, occur in 5–10% of NF1 cases and are often associated with a more severe phenotype including learning disabilities and increased susceptibility to MPNSTs [168, 169]. Intriguingly, such mutations resulting in heterozygous or homozygous loss of *NF1* expression are found to occur more often as sporadic events in AML and ovarian carcinoma, based on cBioPortal data [45]. An *NF1* microdeletion in combination with an abnormal karyotype is an indicator of poor prognosis in AML; 7.6% of ovarian serous cystadenocarcinomas, 2.8% of lung squamous cell carcinomas, 3.3% of glioblastomas and 1.9% of phaeochromocytomas/paragangliomas harboured deletions [45, 82, 84].

*NF1* amplification, and presumably increased neurofibromin expression and hence activity, has been identified in many cancers, including breast (17%), pancreatic (21.5%), uterine endometrial (1.8%) and neuroendocrine prostate cancer (21.5%) [45].

The pathological significance of sporadic *NF1* point mutations, especially putative missense mutations that have been identified in many sporadic tumours, is often unclear. Constitutional *NF1* missense mutations represent about 15% of all *NF1* mutations, but their frequency in sporadic tumours ranges widely from 15 to 71% [24, 25, 45, 72, 106, 143, 167, 170–172]. The characterization of such missense mutations has yielded new insights into the structure and function of neurofibromin. For example, through analysis of missense mutations, the arginine finger loop of the neurofibromin GRD has been found to be crucial for stabilizing the transition state of the GTPase reaction, and many missense mutations in the GRD have been found to exert a significant, pathological effect on Ras activity levels [36, 173, 174].

## Conclusion

Somatic *NF1* mutations are present in tumours associated with NF1 and in a range of sporadic tumours, in different cell types and at various frequencies (Table 1). The frequency and temporal occurrence of somatic mutations and the range of histological types in which they occur therefore imply an important role for neurofibromin function in cancer development and progression. Whilst it is unclear whether the biallelic loss of *NF1* is common or if only heterozygous mutations of *NF1* contribute to tumour progression in sporadic tumours, mouse cells heterozygous for *Nf1* mutations show abnormal growth and invasion [138, 175].

Somatic *NF1* mutations may be critical drivers in multiple cancers as well as contributing to resistance to therapy. The mutational landscape of somatic *NF1* mutation should provide new insights into our understanding of the pathophysiology of cancer.

The introduction of a molecular genomics approach to cancer biology represents a major shift in our approach to the diagnosis and treatment of malignancy. The vast amount of genomic data generated over the last 10 years, which continues to be generated, is providing invaluable insights into the complexities of cancer genome structure, function and evolution. With recent advances in sequencing technology and high-throughput drug discovery, the increasing availability of more sophisticated animal models and the application of the state-of-the-art tumour imaging techniques and the diagnosis and treatment of cancer can only improve. The identification of somatic *NF1* mutations in such a wide spectrum of tumours, including types not associated with NF1, indicates that neurofibromin is likely to play a key role in cancer, far beyond that evident in the tumour predisposition syndrome NF1.

## Abbreviations

ADC: Adenocarcinoma
AML: Acute myeloid leukaemia
AOCS: The Australian Ovarian Cancer Study
CCLE: Cancer Cell Line Encyclopedia
CMML: Chronic myelomonocytic leukaemia
CNS: Central nervous system
COSMIC: Catalogue of Somatic Mutations in Cancer
HGOSC: High-grade serous ovarian carcinoma
HR: Homologous recombination
ICGC: International Cancer Genome Consortium
JMML: Juvenile myelomonocytic leukaemia
MAPK: Mitogen-activated protein kinases
MPNST: Malignant peripheral nerve sheath tumour
NF1: Neurofibromatosis type 1
NSCLC: Non-small cell lung cancer
OSC: Ovarian serous carcinoma
PI3K: Phosphoinositide-3 kinase
RAS-GEFs: RAS guanine nucleotide exchange factors
SCLC: Small cell lung cancer
SqCC: Squamous cell lung cancer
T-ALL: T cell acute lymphoblastic Leukaemia
TCC: Urothelial transitional cell carcinoma
TCGA: The Cancer Genome Atlas
